# A case series of rare pathologies of the aorta and the aortic arch in adolescents and younger adults: Transfer of experience for an individualized approach

**DOI:** 10.3389/fcvm.2022.920614

**Published:** 2022-09-14

**Authors:** Thierry Carrel, Islamjan Sharipov, Adham Jalilov, Juri Sromicki, Paul Robert Vogt

**Affiliations:** ^1^Department for Cardiac Surgery, University Hospital Zürich, Zurich, Switzerland; ^2^Clinic for Cardiac Surgery, Republican Specialized Center for Cardiology, Tashkent, Uzbekistan; ^3^Clinic for Cardiac Surgery, Republican Specialized Center for Cardiology, Qarshi, Uzbekistan

**Keywords:** ascending aorta, aortic arch, surgery, circulatory arrest, cerebral protection, results, adolescents, younger adults

## Abstract

**Objective:**

While aneurysm of the aortic root, interrupted aortic arch, and aortic coarctation are the most frequent aortic diseases in adolescents and younger adults, there are a number of rare pathologies of the thoracic aorta that need individualized treatment.

**Patients:**

We present a small case series of unusual aortic pathologies in patients presenting with a broad spectrum of symptoms: tiredness, dysphagia, dyspnea, arterial hypertension, renal failure, and claudication. (1). Segmental agenesia of the descending aorta. (2). Balanced double aortic arch with complete vascular ring. (3). Right descending aortic arch, left lusorian artery with Kommerell diverticulum. (4). Large patent ductus (2.5 cm) and ventricular septal defect. (5). Aneurysm of the aortic arch in the presence of tuberous sclerosis. (6). Pseudo-aneurysm of the proximal descending aorta following coarctation patch plasty. (7). Supravalvular aortic stenosis combined with severe aortic valve stenosis. (8). Pseudo-aneurysm following ascendens-to-descendens bypass because of recurrent coarctation. (9). Takayasu arteriitis with severe stenosis in the thoraco-abdominal aorta.

**Results:**

The following procedures were performed, using individualized cardiopulmonary bypass, canulation and cerebral protection strategies. (1). Ascending to supraceliac extra-anatomic bypass. (2). Division of the ductus ligament and of the anterior aortic arch distally to the left subclavian artery. (3). Excision of the Kommerell diverticulum and translocation of the left subclavian artery. (4). Patch closure of the aorto-pulmonary window (patent ductus) and closure of the ventricular septal defect. (5). Complete aortic arch replacement combined with debranching of the supra-aortic vessels. (6). Graft interposition of the proximal descending aorta. (7). Enlargement of the ascending aorta and aortic valve replacement. (8). Exclusion of the pseudo-aneurysm, end-to-side graft interposition. (9). Ascending aorta to infrarenal aorta or ascending to bi-iliac artery bypass (planned). All patients were operated on without operative mortality. One patient died in-hospital from pulmonary complications one week after surgery. One patient is still awaiting surgery. All other patients recovered very well from the operation and did not show any residual symptoms.

**Conclusion:**

Rare pathologies of the thoracic aorta in younger patients may cause a broad spectrum of unusual symptoms; in some of them, diagnosis is delayed. Cross-sectional imaging is mandatory for optimal operative planning Surgical treatment can be performed with very satisfying results. The prognosis of these patients is usually favorable following surgery.

## Introduction

While aneurysms of the aortic root in younger patients with connective tissue disease and aortic (re-) coarctation are the most frequent aortic diseases in adolescents and younger adults, there is a number of rare congenital or acquired pathologies that are most often discovered fortuitously. Some of these patients present atypically, with either respiratory symptoms and/or dysphagia because of tracheo-bronchial compression, and receive the correct diagnosis rather late or are misdiagnosed for a long time with asthma, anorexia, and/or other psycho-somatic troubles being suspected. Others may present with hoarseness because of compression or distension of the laryngeal nerve through a large aortic aneurysm. In the presence of concomitant structural heart defects, unusual vascular anomalies are diagnosed earlier in life.

The anatomic details may be highly variable—as may the symptoms—and for this reason, a computer tomography including an angiography or a magnetic resonance imaging are mandatory to most precisely describe the pathology, but also to allow optimal preparation for the surgical intervention. Preoperative echocardiography is also necessary to exclude any additional intracardiac pathology but also to assess cardiac function before a major vascular operation.

In this short paper, we report a series of adolescents and younger patients who received surgery to treat a rare pathology of the thoracic aorta ± aortic arch, with one patient still awaiting surgery. Different techniques have been used, demonstrating that these patients need individualized surgical approaches as well as cardio-pulmonary bypass strategies and cerebral protection management. Written informed consent was obtained retrospectively from the individuals and minors' legal guardians for the publication of any potentially identifiable images or data included in this article, except for 3 patients who were operated on abroad, and from whom data were made non-identifiable.

## Brief description of the cases

### Agenesia of the descending aorta

**Figure 1:**
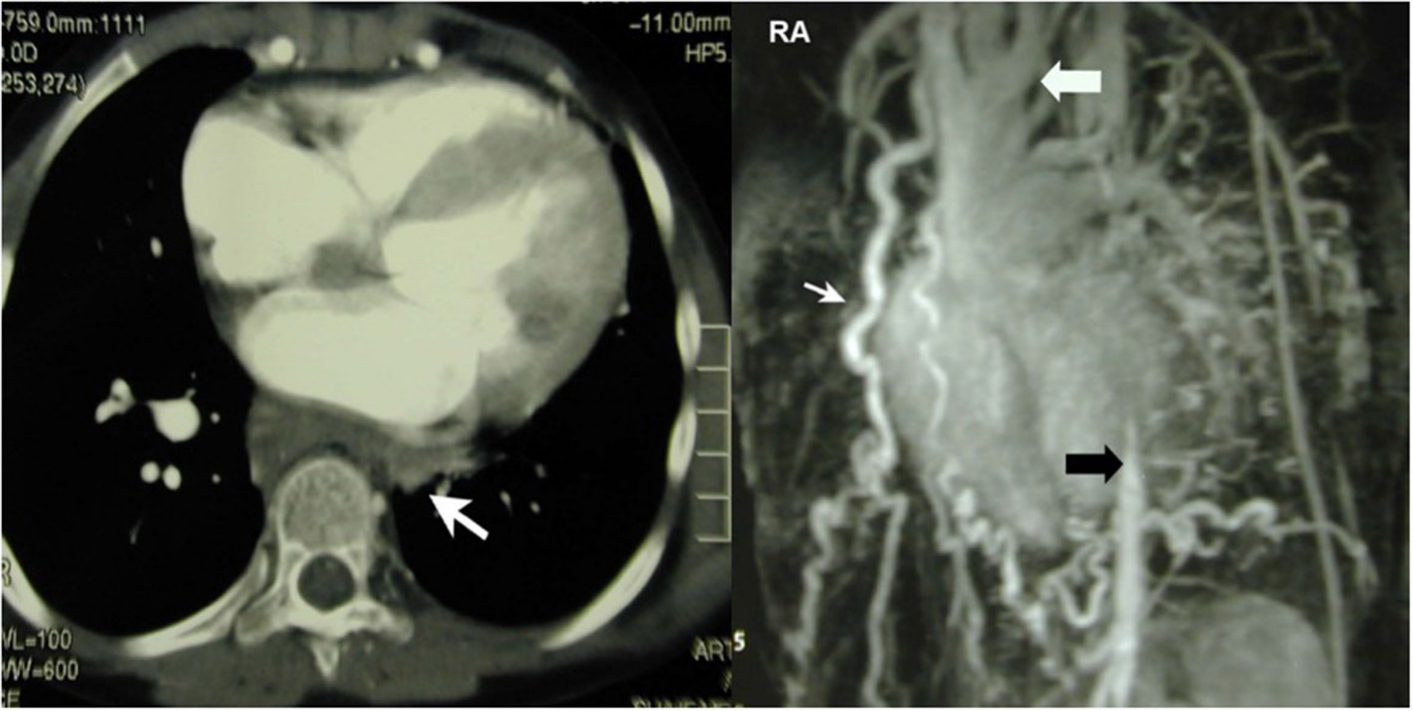
Left: CT-scan showing segmental agenesia (white arrow) of the descending aorta. Right: Huge network of collaterals. The missing segment lies between the distal aortic arch (white arrow) and the thoraco-abdominal junction (black arrow). Reproduced with permission from ICVTS 2003; 2:231–3.

**Figure 2:**
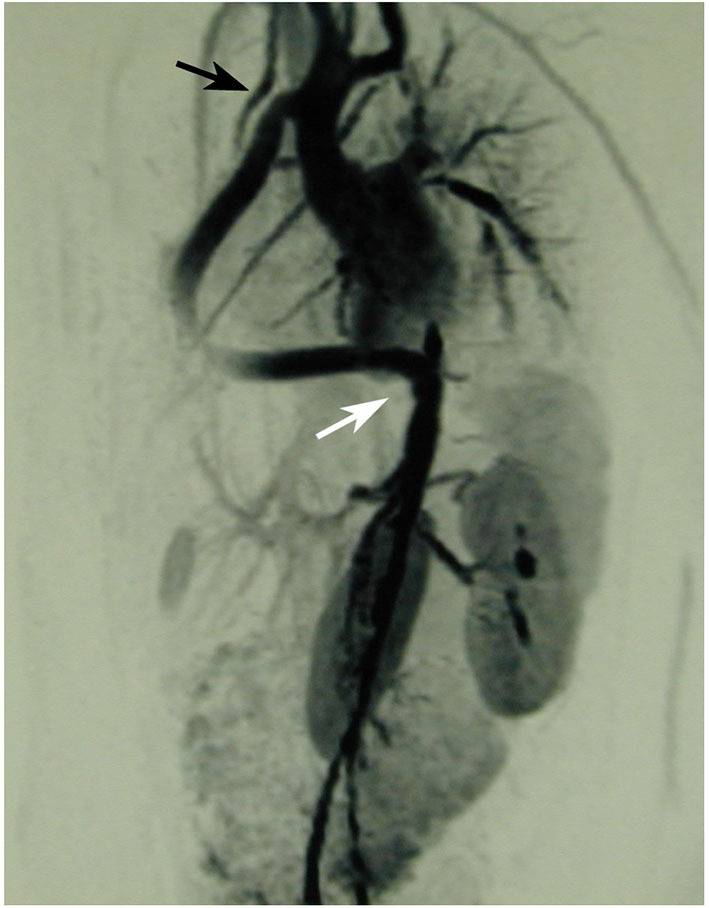
The ascenders-supraceliac bypass is implanted on the right side of the ascending aorta, then in front of the inferior vena cava and exits the pericardial cavity through a limited incision in the posterior pericardium and in the diaphragm to be anastomosed with the supra celiac aorta. Reproduced with permission from ICVTS 2003; 2:231–3.

This girl was aged 12 when she presented with severe arterial hypertension in the upper extremities, a typical sign of restenosis after coarctation repair in the neonatal period. She was put on quadruple anti-hypertensive medication and suffered from claudication of the lower extremities with a limited walking distance of <100 meters. CT-angiography revealed hypoplasia of the distal aortic arch and of the abdominal aorta below the diaphragm and surprisingly a complete absence of the descending aorta in the mid thoracic retrocardiac segment with a multitude of collaterals (Figure 1). Treatment consisted of an extra-anatomic ascending to distal descending aorta bypass *via* sternotomy on the beating heart. The heart was carefully lifted as off-pump coronary bypass grafting and the posterior pericardium was opened. The aorta was prepared at the level of the diaphragm and the end-to-side distal anastomosis performed first with the aorta tangentially clamped. Thereafter the proximal anastomosis was performed on the right lateral side of the ascending aorta. Strict control of the blood pressure is mandatory during clamping, to avoid any localized dissection. This treatment led to almost normalization of the blood pressure and disappearance of leg claudication soon after surgery. Postoperative angio-CT showed a patent graft with unremarkable anastomoses (Figure 2). Reviewing the neonatal hospital chart of this patient did not allow us to determine why the segmental agenesia had not been described earlier.

This case demonstrates how an unexpected and very rare pathology may be misdiagnosed for a long while but finally successfully treated. In the long-term follow-up, the majority of antihypertensive drugs could be stopped, while the patient was maintained on beta-blockers only.

### Balanced double aortic arch with complete vascular ring

**Figure 3:**
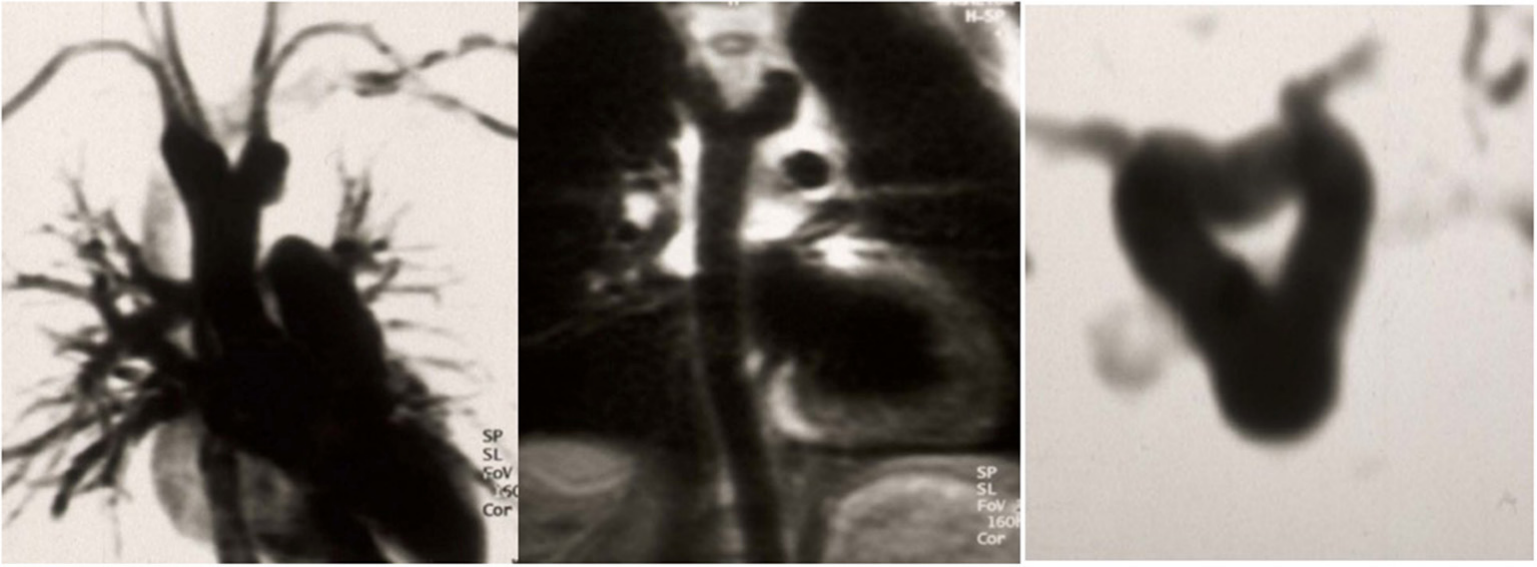
Left: Balanced double aortic arch with each arch giving origin to a subclavian and a carotid artery Middle: confluence in the posterior mediastinum into a single descending aorta. Right: Complete immurement of the trachea and the esophagus within the vascular ring. Reproduced with permission from Schweiz. Med. Wschr. 1999; 129:1722.

A 12-year old girl complained about dyspnea when playing ping-pong at a competition level. Earlier in life, she had a history of bronchial asthma treated with inhalatives and with topical steroids but symptoms did not really improve. Later, she was seen by a pediatric psychiatrist because of dysphagia and symptoms compatible with anorexia. Since no treatment could improve the symptoms, she was consulted by a pediatric cardiologist that performed transthoracic echocardiography. During this examination, a double ascending aorta was suspected and a CT-scan with angiography was recommended. This imaging confirmed the diagnosis of a balanced double aortic arch leading to a complete vascular ring—including the trachea and the esophagus within the narrowed ring. Both arches joined together to form a normal descending aorta at the level of thoracic vertebral body 5/6 (Figure 3). Surgery was performed through a left lateral thoracotomy in the 4th intercostal space and consisted of resection of the ductal ligament, clamping and over-sewing of the anterior, slightly smaller arch distal to the left subclavian artery. Separation of the anterior arch showed impressive decompression of the narrowed space for trachea and esophagus in the operative situs. Postoperative recovery was uneventful and this girl had a normal further development, without any problem during the late follow-up (now 18 years).

### Right descending aortic arch, left lusorian artery with kommerell diverticle

**Figure 4:**
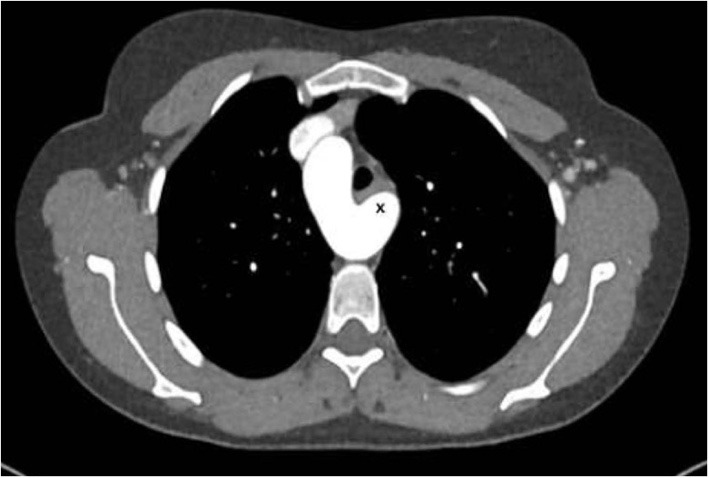
Right descending aortic arch with left arteria lusoria originating from a Kommerell diverticulum (*) with compression of the trachea. Reproduced with permission from Cardiovasc Med: w10132: doi 104414.

**Figure 5:**
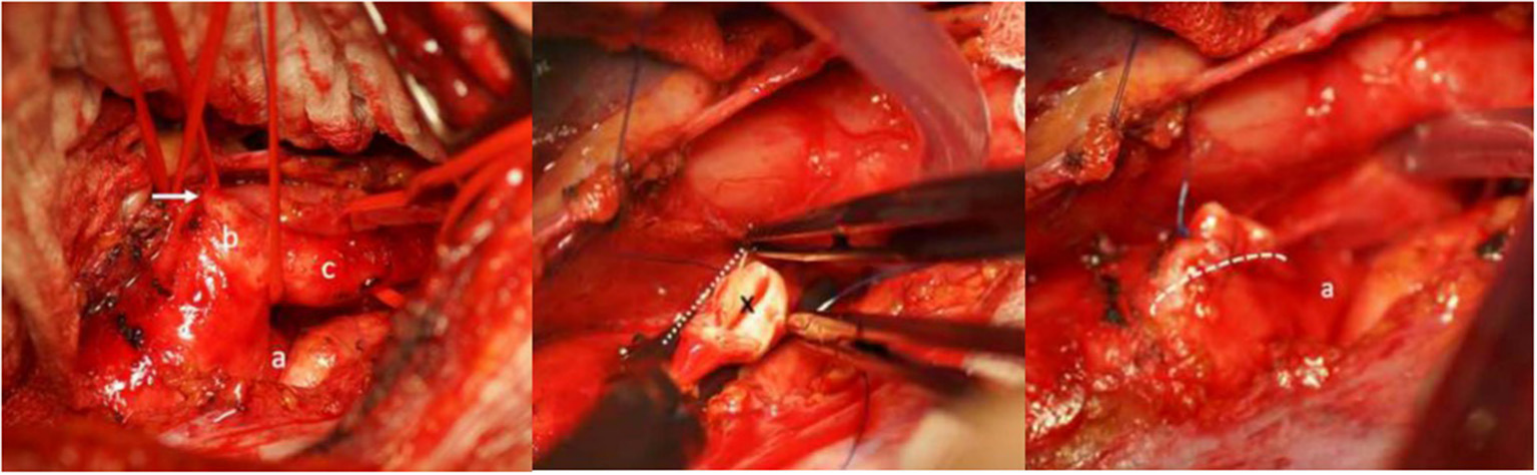
Left: Intraoperative view showing the distal aortic arch (a), the large Kommerell diverticulum (b) and the left subclavian (lusorian) artery (c). The white arrow shows the ductus ligament. Middle: The abnormal left subclavian artery has been transected from the Kommerell diverticulum at its origin (*), the aortic arch is clamped tangentially and the Kommerell diverticulum resected. Right: Picture after resection of the Kommerell diverticulum and longitudinal suture (dotted line) of the aortic arch. A) represents the aortic arch coming from posteriorly. Reproduced with permission from Cardiovasc Med: w10132: doi 104414.

**Figure 6:**
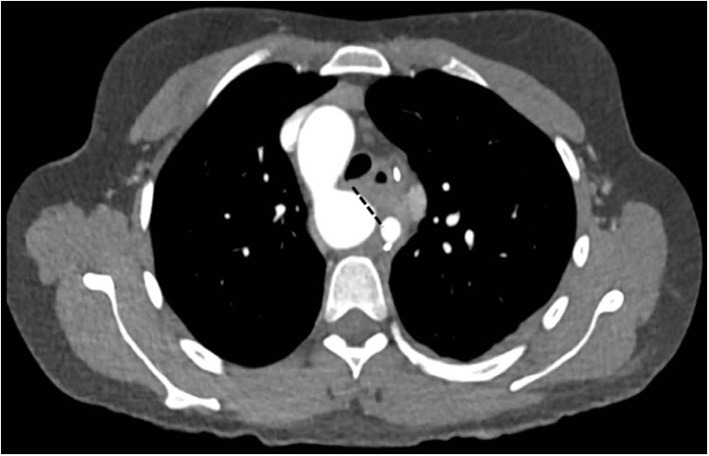
Postoperative CT-angiography after resection of the diverticulum (dotted line). Just on the right is the distally translocated left subclavian artery. Reproduced with permission from Cardiovasc Med: w10132: doi 104414.

A 31-year old woman with a history of bronchial asthma during adolescence was referred because of worsening shortness of breath during minimal exercises at work. A CT-scan was performed and surprisingly showed a right descending aortic arch with an abnormal left subclavian artery (lusorian artery) taking-off from a rather large Kommerell diverticulum that led to a significant compression of the trachea (Figure 4). A bronchoscopy confirmed this observation with a pulsating mass and a “slit-like” narrowing of the trachea. Surgical approach was performed through a left lateral thoracotomy. The ductus ligamentum was divided, and the left subclavian artery was detached from the Kommerell diverticulum and re-implanted more distally into the descending aorta. Finally, the distal aortic arch was clamped tangentially and the Kommerell diverticulum was resected (Figure 5). The intraoperative bronchoscopy at the end of the operation showed a broad opening of the trachea. Postoperative CT-scan did not show any residual tracheal compression (Figure 6).

The patient was controlled 6 months postoperatively and was completely asymptomatic with considerable improvement of the physical performance.

### Descending aorta-pulmonary window (2.5 cm) and ventricular septal defect

A 20–25 year-old female was examined by the cardiologist because of a small restrictive sub-pulmonary ventricular septal defect (dp > 80 mm Hg). Surprisingly, the pulmonary pressure was rather elevated (systolic pressure 90 mmHg) although the defect had always been described as restrictive. Since the patient presented with a 5/6 systolo-diastolic murmur that was auscultable in the back just between the shoulder blades, a CT-scan was performed and showed a 2.5 cm large communication between the proximal descending aorta and the pulmonary artery bifurcation.

**Figure 7:**
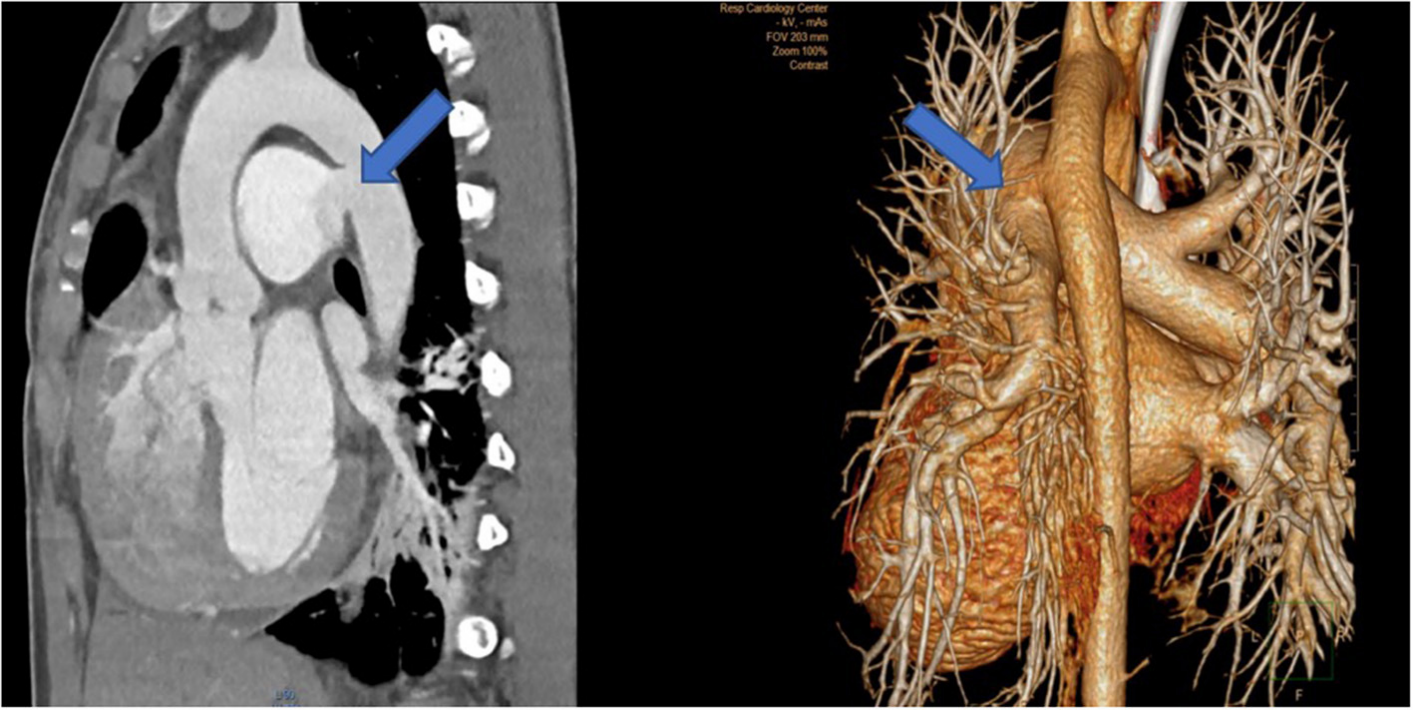
Left: CT-angiography with a large patent ductus with a 2.5 cm confluence into the proximal descending aorta, functionally looking like an aorto-pulmonary window. Right: View in the 3-D reconstruction with the blue arrow demonstrating the site of the communication.

The lesion looked like an aorto-pulmonary window (as seen in the ascending aortic location) but in fact corresponded most probably to an extremely large ductus arteriosus confluence into the aorta (Figure 7). Indication for surgery was discussed since pulmonary artery pressure was not found to be irreversibly elevated.

**Figure 8:**
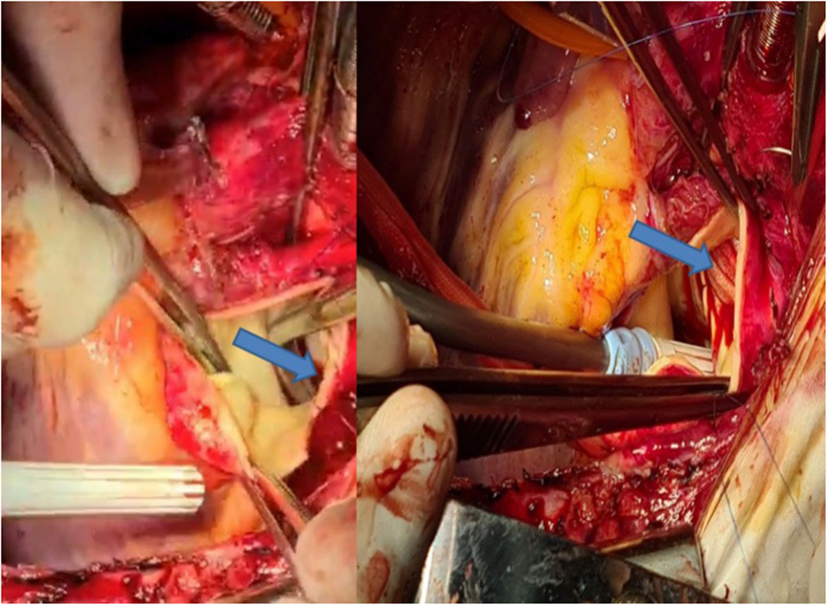
Left: Intraoperative view through the pulmonary artery during circulatory arrest, showing the large communication with the descending aorta (blue arrow). Right: Closure with a vascular patch (blue arrow).

Surgery was performed through a median sternotomy. Prior to aortic canulation, both pulmonary artery branches were encircled with a tape. Thereafter, canulation of the ascending aorta and both vena cava was performed. Just after starting cardiopulmonary bypass, both pulmonary branches were clamped to avoid overfloating of the lungs through the very large aorto-pulmonary connection. At moderate hypothermia (core temperature 30°C), the circulation was briefly (15 min) interrupted, the aortic arch was clamped between the left common carotid and the left subclavian artery, and then antegrade cerebral perfusion was started with a flow of 600 ml at a temperature of 24°C. The ductus was opened longitudinally from its origin at the level of the pulmonary bifurcation until the confluence with the aorta. The large communication was closed using a vascular patch of Dacron with a running suture (Figure 8). We preferred to proceed during cardiac arrest to avoid two clamps close to each other in the operating field during closure of the aortic arch. Thereafter, the ductus was closed at its pulmonary origin, also using a patch. During rewarming, closure of the ventricular septal defect was performed with separate teflon-felt armed U-stitches. Weaning from cardiopulmonary bypass was uneventful and pulmonary pressure decreased to around 40% of the systemic pressure. Further postoperative evolution was uneventful.

### Huge aneurysm of the aortic arch in the presence of tuberous sclerosis

A 23-year old female was followed by neurologists and cardiologists because of a tuberous sclerosis (heterozygote for TSC1: c.989dupT and p.Ser331Glufs^*^10) with multiple but asymptomatic cardiac rhabdomyomas, cortical and periventricular tubera, renal angiomyolipoma and facial angiofibroma.

**Figure 9:**
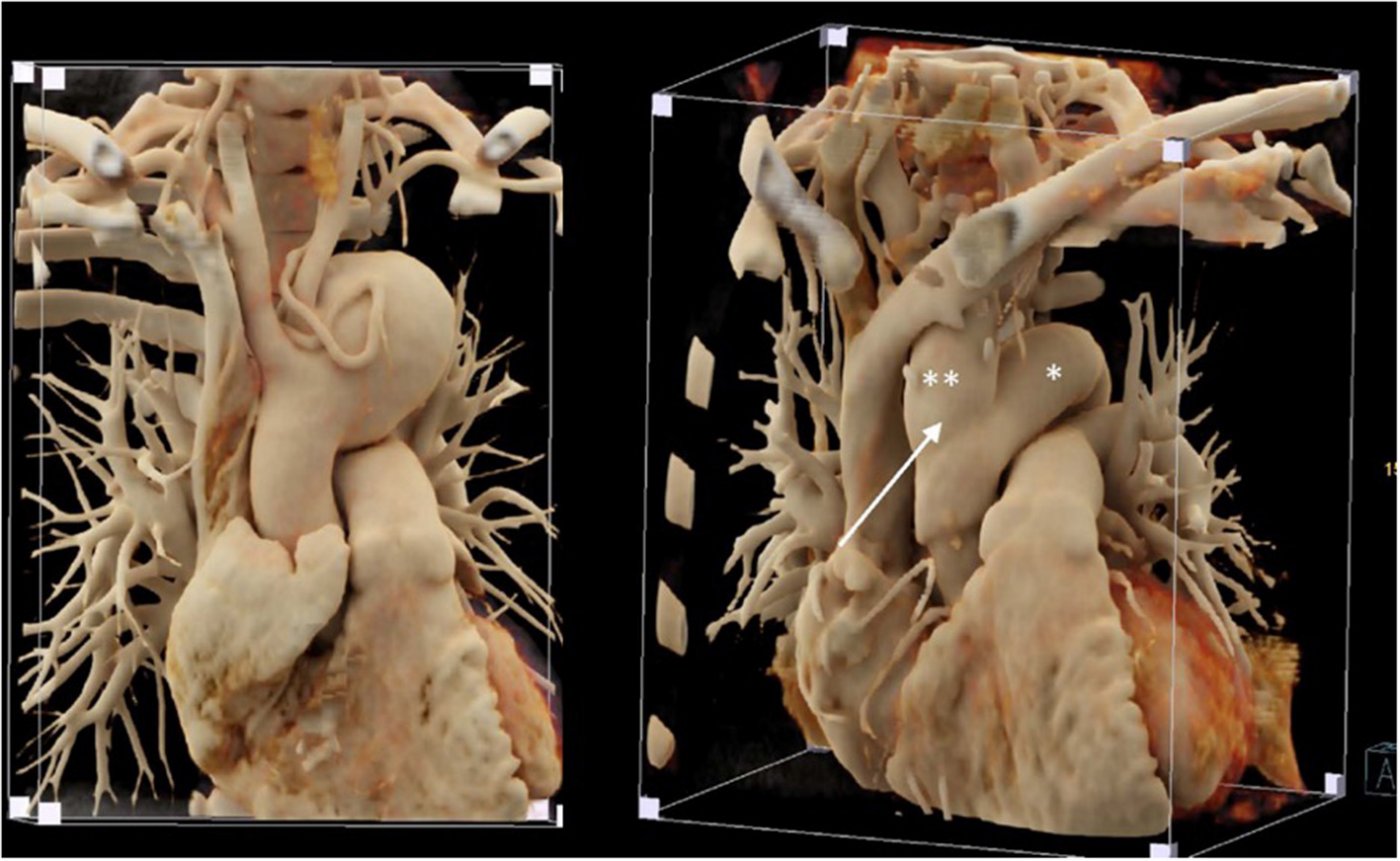
Left: Huge aortic arch aneurysm in a patient with tuberous sclerosis; note the diminutive meandering left subclavian artery around the aneurysm. Right: Postoperative imaging following replacement of the ascending aorta and aortic arch with a 24 mm vascular graft (*) and a separate 20 mm graft to revascularize the innominate artery and the left carotid artery (**) that was implanted in the right lateral ascending graft (white arrow).

Five years before, a slightly dilated aortic arch had been diagnosed. Close monitoring was performed and a huge aneurysm of 6.5 cm in the middle of the aortic arch was suddenly found (Figure 9 left). The patient was completely asymptomatic; the left subclavian artery was very narrowed. Endovascular exclusion was not found to be a good option in this younger adult and surgery was performed through median sternotomy. Using deep hypothermia (core temperature 22°C because of the expected prolonged arrest time), the aortic arch aneurysm was excluded with a 24 mm Vascutek prosthesis, the distal anastomosis performed at the level of the proximal descending aorta first. Then the proximal anastomosis was performed at the level of the mid ascending aorta. The innominate artery and the left common carotid artery were excised as common island and anastomosed to a 20 mm separate vascular graft which was thereafter implanted laterally in the ascending prosthesis (Figure 9 right). The hypoplastic left subclavian artery was ligated. Total circulatory arrest time was 70 min. Rewarming, reperfusion and weaning from cardiopulmonary bypass were uneventful. The patient was discharged 1 week after the procedure without neurological impairment; the pressure difference between both arms was around 30 mm Hg at first postoperative follow-up and the patient had no weakness nor dysaesthesia in the left arm/hand.

### Two cases of huge aneurysm of the proximal descending aorta following different coarctation repair

**Figure 10:**
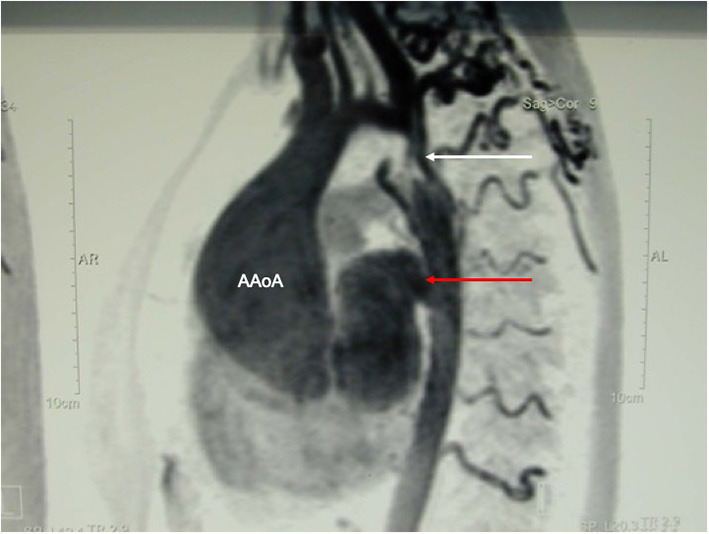
MR-angiography showing a large aneurysm of the ascending aorta (AaoA), a hypoplastic aortic arch and a recurrent stenosis following previous coarctation repair (white arrow) and enlarged intercostal arteries as sign of collateralization. In addition, there was a large pseudoaneurysm at the site of a Dacron patch used for initial coarctation repair (red arrow).

**Figure 11:**
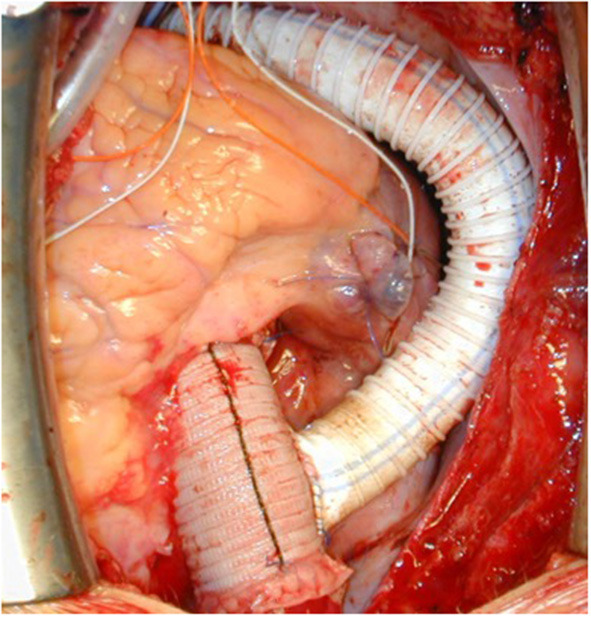
Intraoperative view following replacement of the ascending aorta and ascending-descending bypass implanted laterally in the ascending aortic graft. Reproduced with permission from Ann Thorac Surg. 2008; 85:460–4.

A 23-year old female was referred because of hemoptysis. She had had aortic coarctation repair with a prosthetic patch at the age of 6. Eight years later an ascending-descending bypass was performed because of re-coarctation. At admission, she presented with an ascending aortic aneurysm and a large pseudoaneurysm at the distal anastomosis of the previous extra-anatomic bypass that was itself partially thrombosed. The proximal descending aorta was highly narrowed (Figure 10). A small peripheral aorto-bronchial fistula was suspected as cause of the hemoptysis because it could not be identified during bronchoscopy. Operative repair was performed under double arterial canulation (aortic for the upper body and femoral artery for the lower body perfusion). The procedure was performed through mid-sternotomy and left hemi-clamshell and consisted in double ligation of the descending aorta proximally and distally of the end-to-side previous bypass anastomosis in order to exclude the pseudo-aneurysm. The ascending aortic aneurysm was resected and the aorta replaced with a prosthetic vascular graft and finally, an ascending-descending bypass was performed to restore continuity between the ascending with the distal descending aorta (Figure 11). The suspected site of the aorto-bronchial fistula was addressed by a small wedge-resection and the suture line covered with a intercostal muscle flap. Postoperative recovery was uneventful.

The second case was a 28-year old female who was operated at the age of 10 years and received a patch enlargement as a treatment of aortic coarctation. She did well until the age of 27 when a large pseudo-aneurysm was suspected incidentally on a chest x-ray performed for pulmonary reasons and confirmed by CT-scan. This picture showed in a certain way the challenges of planning surgery with rather simple imaging possibilities in emerging countries. Re-operation was indicated and performed through a lateral thoracotomy under femoro-left atrial bypass and moderate hypothermia (28°C) since a safe clamping of the proximal descending aorta was expected to be difficult. In fact, the aneurysm originated from the suture line of the patch with the native aorta and covered the whole proximal third of the descending aorta. Once the targeted core temperature was reached, the bypass was stopped and the aneurysm opened. Since the period of circulatory arrest was not expected to be prolonged, no additional cerebral protection was planned. Replacement of the proximal descending aorta was performed with the proximal suture line at the level of the left subclavian artery in the open arch technique (circulatory arrest time 14 min) and the distal one at the mid-level of the descending aorta. The vascular graft was approximately 10 cm in length. Weaning from cardiopulmonary bypass was difficult because of a coagulopathy and severe oozing though the graft but inclusion using a pericardial patch allowed the situation to be controlled. The patient was extubated on postoperative day 2. Unfortunately, she had severe pulmonary problems a week later and had to be re-intubated, but unfortunately died 10 days after surgery from respiratory failure.

### Two cases of supra-valvular aortic stenosis combined to severe aortic valve stenosis

Congenital valvular aortic stenosis is pretty common but its association with severe supra-valvular stenosis is much rarer. We present two cases that received a mechanical aortic valve prosthesis with two different aortic enlargement techniques, mainly dependent on the location and extent of the stenosis in the ascending aorta. In the first case, the supra-valvular stenosis was located close to the sino-tubular junction and inspection of the aortic root was difficult because of the narrowing just above the coronary ostia. The 15-20 year-old patient had a history of coarctation repair earlier in life and some signs of re-coarctation with a blood pressure difference between the right and the left arm. In this case, the aorta was incised anteriorly in an oblique way through the sino-tubular junction and the non-coronary sinus was resected.

Following aortic valve replacement, the ascending aorta was replaced with a 26 mm vascular prosthesis including a complete replacement (and thereby enlargement) of the non-coronary sinus through a hemi-Yacoub remodeling technique—since the valve was pseudo-bicuspid (type Sievers I L/R) and the non-coronary sinus accorded for about 50% of the circumference of the aortic root (1).

In the second case—a 13-year old boy—the stenosis of the aorta originated within the aortic root and extent distally upon the cranial part of the ascending aorta. For this purpose, cardiopulmonary bypass was conducted in moderate hypothermia (30°C) to allow sufficient anterior enlargement upon the junction of the ascending aorta to the aortic arch. A xeno-pericardial patch was used: the suture started just at the level of the innominate artery and the patch was given a pantaloon-like configuration proximally, to enlarge both the right and the non-coronary sinus with sparing of the right coronary ostiium (2). The aortic valve was replaced during rewarming. Both cases had an uneventful recovery.

### Large pseudo-aneurysm following ascendens-to-descendens bypass because of recurrent coarctation

**Figure 12:**
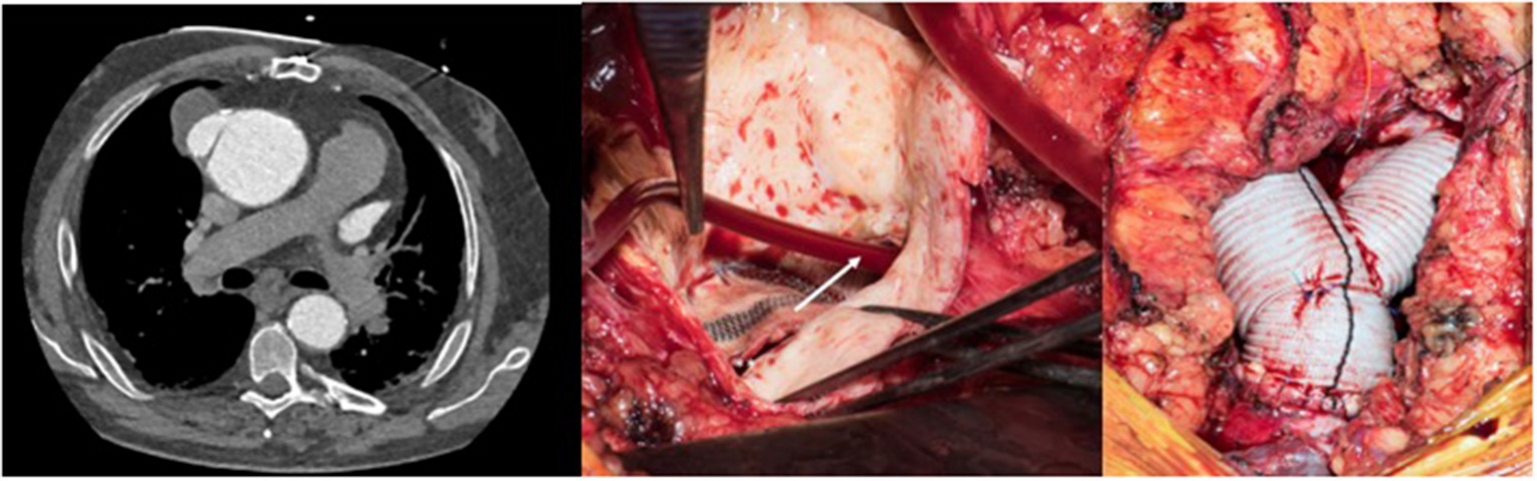
Left: Pseudo-aneurysm at the proximal anastomosis of an ascending-descending bypass performed previously because of a recurrent coarctation. Middle: Intraoperative view after opening of the pseudo-aneurysm with the desinsertion of the anastomosis (*). The extra-anatomic bypass has been blocked proximally to the perfusion canula using a Fogarty catheter (white arrow). Right: View following replacement of the ascending aorta including an open arch distal anastomosis during circulatory arrest with bilateral antegrade cerebral perfusion and proximal extension of the extra-anatomic bypass to allow tensionless re-implantation in the ascending graft.

A 45-year old patient had a complex medical history due to a Shone syndrome, including coarctation repair with end-to-end anastomosis in the neonatal period followed by aortic valve replacement with a mechanical prosthesis and ascendens-to-descendens bypass at the age of 22 years because of a recurrent coarctation. Following this second surgery, the patient was doing fine until a large pseudoaneurysm was discovered at the proximal anastomosis of the extra-anatomic conduit on the ascending aorta (Figure 12 left). The patient suffered from unspecific thoracic pains and surgery was decided upon. The operation was performed after canulation of the ascending aorta for the upper body perfusion and canulation of the extra-anatomic conduit for the lower body perfusion while a two-staged right atrium canula was used for venous return. Following cross-clamping of the ascending aorta and blockage of the extra-anatomic conduit with a Fogarty catheter, the aneurysm was opened and cardioplegia was administered directly into the coronary ostia. A dehiscent anastomosis was found (Figure 12 middle). No signs of infection were found. The ascending aorta was replaced with a short graft and the most proximal part of the extra-anatomic bypass was replaced with a new vascular graft to allow a tension-free anastomosis (Figure 12 right). Postoperative course was uncomplicated except for the difficult psychic situation of the patient (already known preoperatively), which remained demanding.

### Two cases of Takayasu arteriitis with several stenosis in the distal aortic arch, in the descending and thoraco-abdominal aorta

**Figure 13:**
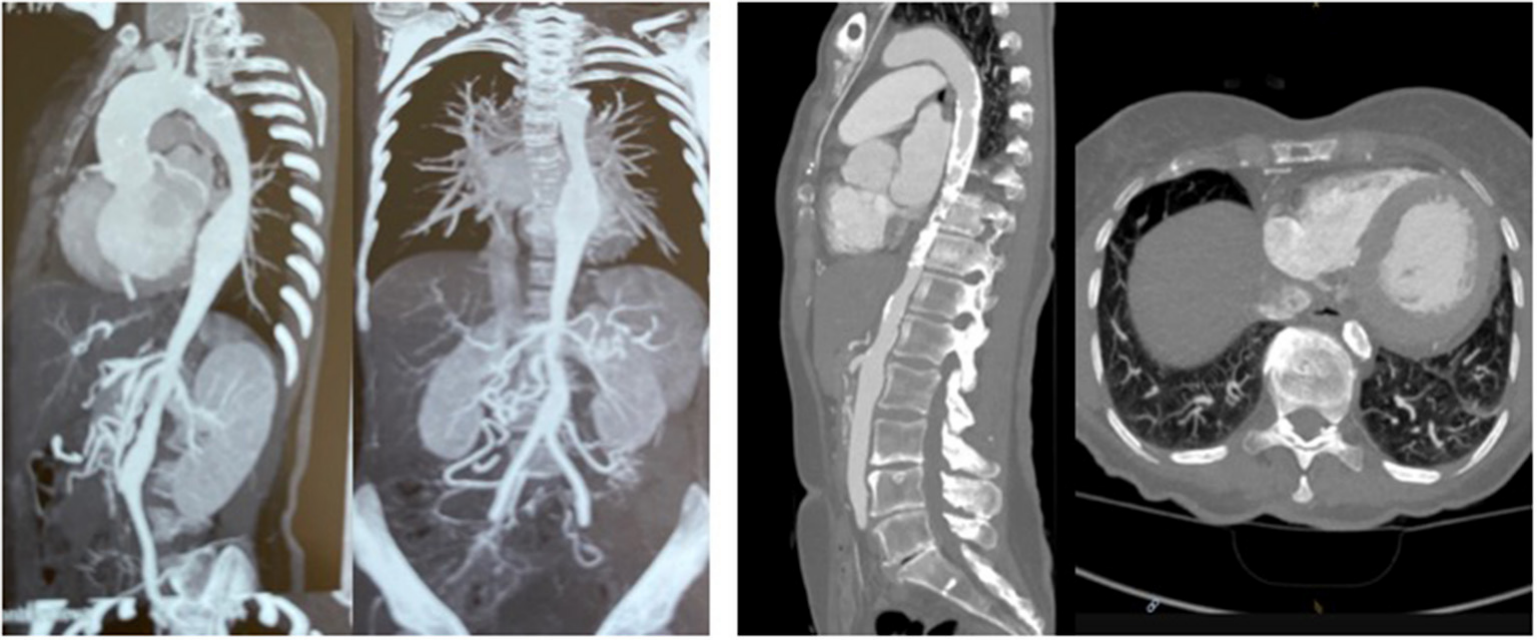
Left (2 pictures): CT-angiogram of a young patient with Takayasu aortitis with multiple stenoses in the thoracic and abdominal aorta and occlusion of the iliac arteries scheduled for an descending to bi-iliac extra-anatomic bypass. Right (2 pictures): CT-angiogram of a 45 yr-old female with severe calcific narrowing of the descending aorta as a result of a Takayasu more than 25 years before. Endovascular procedure was thought to be not ideal in this case.

The first patient is a 15–20 year-old female who suffered from increasing claudication symptoms that appeared after a walking distance of <50–100 meters. Suspicion of Takayasu arteriitis was made in the past and clinical examination revealed arterial hypertension of the upper extremities but no pulse at the femoral arteries and distally of them. CT-scan with angiography showed several narrowing at the level of the descending thoracic but also of the abdominal aorta (Figure 13 both left). Since the contrast agent seemed to stop at the level of the external iliac arteries, a direct angiography was planned but the catheter could not be introduced, neither into the radial nor into the femoral arteries for selective injections. At that stage we recommended an extra-anatomic (subcutaneous) thoraco-bifemoral bifurcated graft and this procedure will be performed as soon as possible.

**Figure 14:**
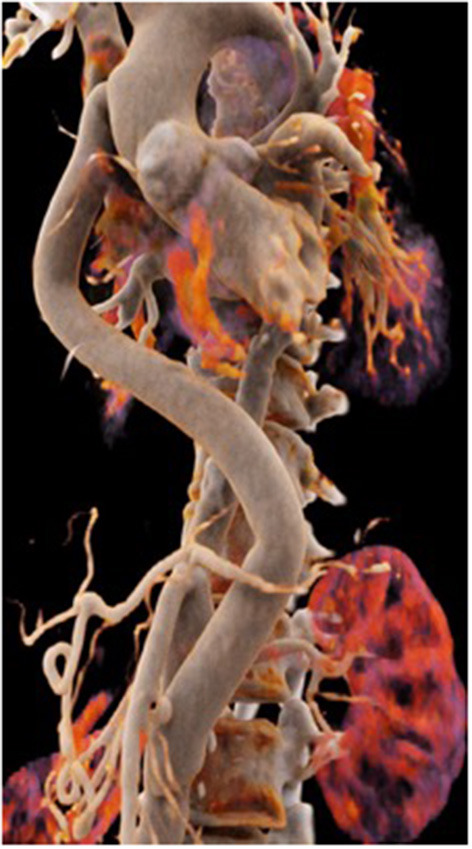
Postoperative 3-D reconstruction of the CT-angio following ascending to infrarenal bypass in the patient with very calcified and narrowed descending aorta following Takayasu disease.

The second patient is a 45-year old female known for a Takayasu arteriitis who suffered from drug-resistant arterial hypertension, renal failure and symptoms of claudication Fontaine stage IIb-III with a walking distance of <100 m and sometimes pains at rest. She presented with a localized severe calcific stenosis of the descending aorta with a resting lumen of 3–4 mm at the narrowest site (Figure 13 both right). She was originally referred for evaluation of an endovascular approach but the interventional radiologist denied this possibility. Finally, she received an extra-anatomic ascending-to-infrarenal aortic bypass. A 16 mm vascular graft was anastomosed on the beating heart at the right lateral level of the tangentially clamped ascending aorta. The graft was then brought to the right close to the right atrium and passed through the diaphragm, then through the bursa omentalis to the retroperitoneum and the infrarenal aorta where the distal anastomosis was constructed approximatively 4–5 cm above the aorto-iliac bifurcation. Postoperative course was uneventful and symptoms as well as the arterial hypertension greatly improved soon after surgery. CT-scan demonstrated the correct position of the extra-anatomic graft (Figure 14).

## Comment

This limited series demonstrates the diversity of rare pathologies of the thoracic aorta—including the aortic arch and the descending part—that may be encountered in adolescents and younger adult patients in the western world but also in emerging countries. In a substantial proportion of these patients, the diagnosis is delayed because symptoms are atypical (3). This report should encourage practitioners to proceed early with cross-sectional imaging including either computed tomography and/or magnetic resonance imaging when dyspnea, dysphagia, uncontrollable hypertension, or other rare symptoms like thoracic pains are encountered in adolescents and younger adult patients with otherwise an unspectacular medical history (4). On the other side, we would like to emphasize the importance of close monitoring in those patients that received a surgical and/or endovascular aortic treatment in the early years of life since potentially life-threatening conditions may develop in the long-term after previous cardiovascular interventions (5). In these patients, symptoms like dyspnea (without any cardiac nor pulmonary cause), dysphagia, hoarseness or thoracic pains, should lead to suspicion of aneurysms or pseudoaneurysms.

The surgical approach is performed through a median sternotomy or left lateral thoracotomy for the large majority of cases. Occasionally combining a mid-sternotomy with a left hemi-clamshell approach gives a comfortable approach to the whole descending aorta. Management of cardiopulmonary bypass and the need for cerebral protection has to be individualized while a large experience from routine but also more complex adult aortic (and aortic arch) cases requiring particular surgical techniques is of great advantage to deal with such cases, especially when operating in emerging countries (6–8).

Some of these cases were treated in Usbekistan within a cooperation project between the Republican Specialized Center for Cardiology in Tashkent and Qarshi, the Department of Cardiac Surgery at University Hospital in Zürich and the Foundation for Children Heart (Corelina), and the EurAsia Heart Foundation which has been involved in the field for more than 20 years.

Cases are discussed and prepared together, defining the optimal perfusion strategy with the surgeon, the anesthesiologist, and the perfusionist. Bail-out options are discussed in the team to avoid surprises at any time during the surgery. This is why such cases should be operated on by surgeons with an extensive experience in complex thoracic aortic surgery, including arch surgery (during beating heart or circulatory arrest), hypothermic circulatory arrest, and different strategies of cerebral protection. During missions in centers with less experience to treat severe aortic pathologies, we prefer deep hypothermia (20–24°C) in case of any intervention at the level of the aortic arch; this is the simpler technique, it does not need additional material like perfusion catheters for the supra-aortic branches and has been proved to be safe for up to 45 min. Otherwise we usually use moderate hypothermia (26–30°C) and bilateral selective antegrade cerebral perfusion.

Since thoracic aortic cases, especially those involving the aortic arch, may be technically demanding, they should not be treated during what Corno not inappropriately named “surgical safaries” but preferentially during specialized educational missions in those institutions abroad where programs can be established in the long-term (9).

The success of such programs cannot be defined only by the number of cases that are operated upon and the level of technical difficulty that is encountered, but finally by the results and by the transfer of knowledge which will allow the local teams to progressively operate on such pathologies on their own.

Altogether, surgical results following these rare and technically demanding cases are promising, the survival rate is excellent, and clinical improvement in terms of the disappearance of symptoms is observed soon after the intervention.

## Data availability statement

The datasets for this article are not publicly available due to concerns regarding participant/patient anonymity. Requests to access the datasets should be directed to the corresponding author.

## Ethics statement

Ethical review and approval was not required for the study on human participants in accordance with the Local Legislation and Institutional Requirements. Written informed consent for the operative procedure and to participate in this study was provided by the participants' legal guardian/next of kin.

## Author contributions

TC, IS, AJ, JS, and PV contributed to conception and design of the study. TC organized the database and wrote the first draft of the manuscript. TC, IS, AJ, and JS operated patients. All authors contributed to manuscript revision, read, and approved the submitted version.

## Conflict of interest

The authors declare that the research was conducted in the absence of any commercial or financial relationships that could be construed as a potential conflict of interest.

## Publisher's note

All claims expressed in this article are solely those of the authors and do not necessarily represent those of their affiliated organizations, or those of the publisher, the editors and the reviewers. Any product that may be evaluated in this article, or claim that may be made by its manufacturer, is not guaranteed or endorsed by the publisher.
